# Digital Health Secondary Prevention Using Co-Design Procedures: Focus Group Study With Health Care Providers and Patients With Myocardial Infarction

**DOI:** 10.2196/49892

**Published:** 2023-10-30

**Authors:** Melissa Louise Pelly, Farhad Fatehi, Danny Liew, Antonio Verdejo-Garcia

**Affiliations:** 1 Turner Institute for Brain and Mental Health School of Psychological Sciences Monash University Melbourne Australia; 2 School of Public Health and Preventive Medicine, Monash University Melbourne Australia

**Keywords:** co-design, digital health, myocardial infarction, qualitative, participatory, mobile health

## Abstract

**Background:**

Myocardial infarction (MI) is a debilitating condition and a leading cause of morbidity and mortality worldwide. Digital health is a promising approach for delivering secondary prevention to support patients with a history of MI and for reducing risk factors that can lead to a future event. However, its potential can only be fulfilled when the technology meets the needs of the end users who will be interacting with this secondary prevention.

**Objective:**

We aimed to gauge the opinions of patients with a history of MI and health professionals concerning the functions, features, and characteristics of a digital health solution to support post-MI care.

**Methods:**

Our approach aligned with the gold standard participatory co-design procedures enabling progressive refinement of feedback via exploratory, confirmatory, and prototype-assisted feedback from participants. Patients with a history of MI and health professionals from Australia attended focus groups over a videoconference system. We engaged with 38 participants across 3 rounds of focus groups using an iterative co-design approach. Round 1 included 8 participants (4 patients and 4 health professionals), round 2 included 24 participants (11 patients and 13 health professionals), and round 3 included 22 participants (14 patients and 8 health professionals).

**Results:**

Participants highlighted the potential of digital health in addressing the unmet needs of post-MI care. Both patients with a history of MI and health professionals agreed that mental health is a key concern in post-MI care that requires further support. Participants agreed that family members can be used to support postdischarge care and require support from the health care team. Participants agreed that incorporating simple games with a points system can increase long-term engagement. However, patients with a history of MI emphasized a lack of support from their health care team, family, and community more strongly than health professionals. They also expressed some openness to using artificial intelligence, whereas health professionals expressed that users should not be aware of artificial intelligence use.

**Conclusions:**

These results provide valuable insights into the development of digital health secondary preventions aimed at supporting patients with a history of MI. Future research can implement a pilot study in the population with MI to trial these recommendations in a real-world setting.

## Introduction

### Myocardial Infarction

Myocardial infarction (MI) is the leading cause of morbidity and mortality globally [1]. Experiencing an MI increases the likelihood of a subsequent MI, resulting in 30% higher mortality rates than the general population [1]. Secondary prevention strategies can be implemented to support patients with a history of MI, particularly when regular contact with their health care provider is no longer feasible. These strategies have the potential to facilitate patients with a history of MI self-manage their condition in alignment with health providers’ recommendations and promote positive, heart-healthy behaviors. Cardiac rehabilitation programs are a traditional form of secondary prevention used to encourage positive behavior change after MI and are found to reduce readmission and mortality [2,3]. However, the attendance and engagement of patients with a history of MI in these programs is low [4]. Studies have identified several barriers to cardiac rehabilitation, including concerns about engaging with the content, travel time, fees, or conflicting schedules [4-6]. Therefore, other forms of secondary prevention are needed to promote the lifestyle changes required to manage MI risk factors.

Key protective factors for MI include smoking cessation, increased physical activity, and a healthy diet [7]. However, a global study on cardiovascular disease using data spanning between 2003 and 2009 from 153,996 adults determined that only 4.3% of adults with cardiovascular disease adopted all 3 positive lifestyle behaviors [8]. To substantiate this, an 11-year longitudinal study found that 79% of MI events could be prevented in men who adhere to 5 protective factors: a healthy diet, reasonably low alcohol consumption, smoking cessation, high physical activity, and the absence of a high waist circumference [7]. Despite its clear importance, there is a markedly low level of adherence to protective factors in the cardiovascular population, indicating that other efforts need to be made to assist patients with a history of MI in changing negative health behaviors.

### Digital Health

Digital health has become an increasingly feasible modality for implementing behavior change support in a clinical population in a cost-effective manner [9,10]. It can directly address the shortcomings of traditional cardiac rehabilitation. Namely, ensuring that content is engaging with each user, eliminating the need for travel, reducing fees, aligning with patient schedules, and being easy and accessible so that patients can easily implement lifestyle changes [4-6]. Digital health technologies have been widely supported among scientific and health care communities, with the American Heart Association encouraging the use of mobile health (mHealth) for cardiovascular disease prevention [11]. Their stance was based on multiple randomized controlled trials using mHealth, which resulted in weight loss, physical activity, smoking cessation, blood glucose management, hypertension management, and lipid management [11]. Digital health has been successfully used to reduce cardiovascular disease risk factors and outcomes, with secondary preventions leading to a 40% reduction in the relative risk of cardiovascular disease outcomes and reduced morbidity and mortality [12]. Specifically, digital health secondary preventions led to significant reductions in systolic blood pressure, reduced antiaggregant medication nonadherence by 69%, and reduced rehospitalization by 55% compared with standard care [13]. This risk reduction is greater than that of other common preventive measures, such as statins, aspirin, and blood pressure reductions with β-blockers [12]. Ultimately, the all-cause mortality rate is reduced by 49% when comparing those who used digital health secondary preventions compared with standard care, showing the utility of digital health in cardiovascular disease secondary prevention [13].

mHealth, a subset of digital health, is a highly used health care tool owing to its intuitive implementation, with most people in Western countries owning an mHealth device [14]. mHealth interventions have been shown to significantly improve lifestyle cardiovascular risk factors, including improvements in systolic-diastolic blood pressure levels, smoking cessation, medication adherence, BMI, patient satisfaction, and quality of life after 1 year compared with usual care [15,16]. mHealth is a useful tool as patients with a history of MI carry mobile phones throughout the day, making daily mHealth interventions possible, such as implementing timely SMS text messages [17-19]. SMS text messaging interventions can provide advice, motivational reminders, and lifestyle behavior change support and have been found to decrease low-density lipoprotein cholesterol levels, systolic blood pressure, BMI, and smoking and improve physical activity and medication adherence [17-19]. Therefore, there are marked clinical and behavioral indicators showing utility in digital health and mHealth approaches [20], yet they have not been widely adopted [21,22].

Key barriers to the adoption of digital health are a lack of technology usability and user involvement during the design of the technology [21]. One way to improve technology usability is to use machine learning algorithms, which aim to learn from existing data and adapt their output using mathematical models [23]. Machine learning is based on big data (which are becoming increasingly available using mobile tracking) and can be used to predict individual health behavior and tailor the technology to the life and context of patients with a history of MI [24]. In addition, designing digital health technology with the advice of end users can mitigate these barriers by gaining insight into how users would practically use technology to support their health needs [25].

### Co-Design Research

This approach to intervention development is known as co-design or participatory research [22]. The following co-design principles allow researchers to understand the needs of the target population, enhance communication and cooperation between stakeholders, and increase user satisfaction and loyalty [26]. Each of the parties involved in the co-design should provide valuable insights into their expertise. The early stages of co-design are used extensively in novel intervention development [27]. The architecture of these early stages of co-design is mostly studied using focus group discussions (FGDs) and interviews with the stakeholders of the solution, that is, the user and health professionals (HPs) [27,28].

### This Study

Our study, deemed MiSmartHeart, aimed to identify the core needs of patients with a history of MI in the context of the prevention of subsequent MI events and explain how these can be addressed using features of a digital health solution, as identified by both patients with a history of MI and HPs.

## Methods

### Design

The MiSmartHeart study used a qualitative and iterative approach to refine the essential components of MI secondary prevention informed by end users. This study uses similar co-design applications as those reported in the specialized co-design literature [29,30]. For instance, this study involves iteratively obtaining opinions and advice from multiple stakeholders using FGDs and interviews across multiple rounds of discussion, which leads to mock-ups of the solution [27]. These rounds of discussion can refute, refine, build on, or confirm advice from the previous rounds. This study comprised 3 iterative phases deployed via 3 rounds of FGDs. The 3 rounds are defined as follows:

Round 1 was titled “consumer needs.” In this round, we attempted to determine what the overall population needs are by discussing real-world issues with involved parties, that is, patients with a history of MI and HPs. Round 1 was broad and exploratory, and the findings were consolidated by identifying overarching unmet needs. The results of this round led to an initial conceptual design developed by the research team.

Round 2 was titled “desired functionalities,” and it involved obtaining more information about the core unmet needs identified in round 1 and methods to address these needs. Participants were shown visual conceptual designs created based on the advice from round 1. The research team explained to participants that these designs were intended to relay core concepts of possible digital health features identified in round 1, rather than depicting a visual mock-up of a designed intervention. Round 2 was more structured and less broad than round 1, as the discussion focused on elaborating on a few key unmet needs identified in round 1. The research findings were consolidated by identifying categories and subcategories and drawing conclusions into a simulated design. The results of this round led to the creation of an in-depth simulation displaying each feature of the proposed app to be discussed in the next round of FGDs.

**Figure 1:**
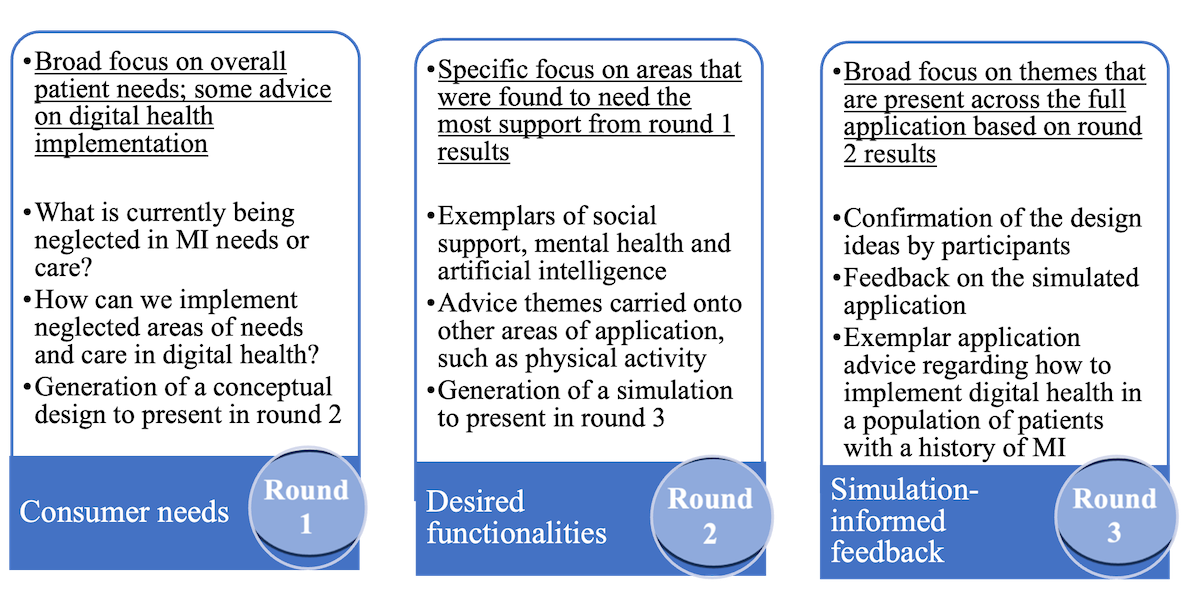
Anticipated outcomes for each round of focus group discussions. MI: myocardial infarction.

Round 3 was titled “simulation-informed feedback,” and it involved providing an example simulation to generate new ideas to address the problem and to gain additional feedback. The results of this round provided a comprehensive overview of the additional features and functions needed in a self-management app to support patients with a history of MI. These insights are broad and not bound to the key unmet needs elaborated on in round 2. These insights can be integrated into a fully digital health intervention. The key anticipated outcomes for each round have been described in Figure 1.

### Participants

A total of 38 participants were recruited across the 3 focus group rounds, with 11 participants recruited in multiple rounds. Inclusion criteria were (1) aged ≥18 years and (2) self-reported experience of an MI for which they were subsequently hospitalized or self-reported involvement in the care of patients with a history of MI. Participants were excluded if they could not speak English, did not reside in Australia, or did not own a device to allow communication with the research team (ie, mobile phone or computer).

Round 1 included 8 participants (4 patients with a history of MI and 4 HPs). As this round was primarily intended as an initial scope to enable further exploration in future rounds, this sample size is within acceptable standards for the exploratory stages of co-design according to the specialized literature [28]. Round 2 included 24 participants (11 patients with a history of MI and 13 HPs). Round 3 included 22 participants (14 patients with a history of MI and 8 HPs). As rounds 2 and 3 required more idea generation and evaluation, we aimed to recruit a larger number of participants. In round 3, we intended to recruit more patients with a history of MI than HPs. This is because patients with a history of MI would be the primary consumer of the digital health intervention; therefore, they would be best able to advise and evaluate the final simulated prototype. Within each round, there were no repeat interviews or FGDs. Considering repeated participation across rounds, we engaged with the participants a total of 54 times. Participants’ demographics were explained across the 3 rounds (Table 1).

**Table 1:** Participant information across 3 rounds of focus group discussions.

Participant types and characteristics		Round 1 (consumer needs)	Round 2 (desired functionalities)	Round 3 (simulation-informed feedback)
**Patient**				
	Age (y), mean (SD)	40.75 (13.36)	51.82 (12.43)	49 (10.59)
	Female sex, n/N (%)	3/4 (75)	5/11 (45)	7/14 (50)
	Retained from previous round	N/A^a^	2 patients retained from round 1	5 patients retained from round 1 or 2
	Highest education	2 certification or accreditations 3 or 4 2 diploma or advanced diploma	5 certification or accreditations 3 or 4 2 diploma 2 bachelor 1 postgraduate 1 no answer	4 certification or accreditations 3 or 4 4 diploma 2 no answer 1 postgraduate 1 bachelor 2 high school
	Race or ethnicity	3 Australian participants 1 Asian participant	10 Australian participants 1 other participant	11 Australian participants 3 other participants
**HP^b^**				
	Female sex, n/N (%)	3/4 (75)	9/13 (69)	7/8 (88)
	Retained from previous round	N/A	4 HPs retained from round 1	5 HPs retained from round 1 or 2
	Profession	1 GP^c^ 1 physiotherapist 1 aged care emergency attendant 1 rehabilitation consultant	5 cardiac rehabilitation specialists 4 cardiac nurses 2 GPs 1 physiotherapist 1 aged care emergency attendant	4 cardiac rehabilitation specialists 4 cardiac nurses

^a^N/A: not applicable.

^b^HP: health professional.

^c^GP: general practitioner.

We intended to recruit both new and existing participants in the study for various reasons. First, existing participants were needed in the study to amend or confirm that our representation of their advice from the previous round was accurate and achieved its intended purpose. However, new participants were recruited to reduce the potential for participant bias, that is, confirming the outcome because it is based on their advice rather than critiquing the outcome of their advice. New participant recruitment was also needed for a greater likelihood of generating new ideas and concepts that were not explored in the previous rounds. Amending and confirming previous participant advice with newly recruited participants provided more reliability in that the shared ideas represent opinions held by this population.

### Procedure

This study was conducted between November 2020 and December 2021. The researchers conducted data analysis and obtained the results after each round to implement insights into the following round.

Researchers conducted FGDs and qualitative interviews with patients with a history of MI and HPs (eg, general practitioners, nurses, and cardiac specialists). Participants were people living in Australia who were aged ≥18 years. Eligibility criteria was as follows: the patients with a history of MI must have a history of hospitalization for MI and HPs must have experience treating patients with a history of MI. Participants were consecutively sampled with either web-based advertisements on various cardiology-focused Facebook pages and social media websites, or in person, with flyers delivered to cardiology clinics and assisted living villages in Victoria, Australia. Advertisements prompted participants to fill out an expression of interest form, after which the researchers emailed the participants. Participants had no prior relationship with the researchers but understood that the study was a part of PhD research concerning behavior change.

Semistructured FGDs and interviews were also conducted. FGDs were organized based on availability, with FGDs preferred over interviews to allow the exchange of ideas between participants. If the participant schedules did not align or they did not feel comfortable speaking with other participants, an interview was conducted instead. FGDs and interviews were delivered on either Zoom (Zoom Video Communications), a popular videoconference system, or a phone call with the participant if they could not use Zoom. All attendees in these FGDs and interviews were recruited as participants for this study. A question guide was created based on the research team’s expert knowledge of digital health, behavior change, and cardiovascular disease. The question guides from rounds 2 and 3 were created considering the advice provided from the previous round, with an emphasis on exploring the previously mentioned topics with more granularity. The full question guides outlining discussion topics for each round are available (Multimedia Appendix 1). FGDs were held with a maximum of 5 people in the session, along with 2 interviewers (duration: 60 min). Most focus group sessions included 2 or 3 participants. The interviews consisted of 1 patient with a history of MI or an HP, along with 2 interviewers (duration: 40-60 min). One male interviewer had an extensive background in digital health for chronic disease (FF); the other female PhD student had experience in clinically interviewing cardiology patients in a hospital setting (MLP). Together, these interviewers conducted FGDs and interviews across all rounds. Recruitment continued until the researchers agreed that data saturation for that round was reached. Audio and video were recorded on Zoom with the participants’ consent and then transcribed. Transcripts and results were not sent to participants for confirmation.

### Ethical Considerations

The Monash University Human Research Ethics Committee approved this study (reference: 25035, 22/7/2020), and all participants provided informed consent. Participants were reimbursed with an Aus $30 (US $19.12) shopping e-voucher. All data was deidentified upon data analysis and writeup.

### Data Analysis

Our descriptive data analysis involved a flexible approach, which was found to be effective in obtaining rich data [31]. The qualitative methods use the tenants of naturalistic inquiry, with a primary interest in studying humans in their natural state without the constraints of preexisting theoretical underpinnings [32]. For all transcribed focus group and interview discussions, data were extracted into NVivo (version 20.5; Lumivero). Here, participants were separated into “patients with a history of MI” and “HP” to establish initial codes based on the data. Once codes were created, categories that encompassed the codes for each patients with a history of MI and HP group were defined.

Multiple iterations were performed to determine which groups of categories were the most representative of the data. Each category consisted of subcategories (grouped codes under a category). When a consensus among the 2 researchers (ie, the first and second authors) was reached, key data were extracted into an Excel (Microsoft Corporation) sheet based on these key categories and subcategories. From here, categories were sorted from the most common to least commonly mentioned, which was used to prepare the results. The frequency of each category for each patients with a history of MI and HP group, along with illustrative quotes from the participants, was included.

## Results

### Round 1: Consumer Needs

#### Overview

**Figure 2:**
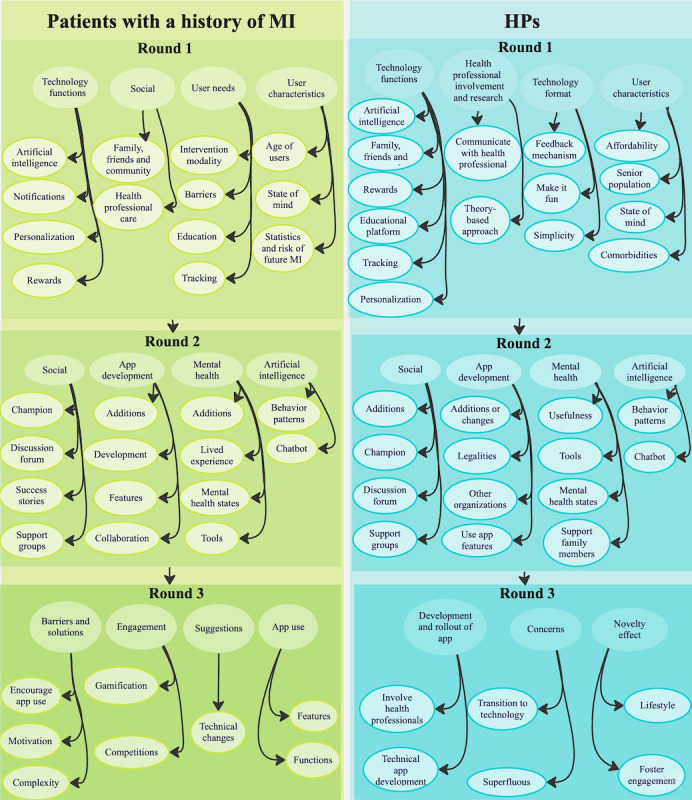
Categories and subcategories in rounds 1 to 3 for patients with a history of myocardial infarction (MI) and health professionals (HPs).

Discussions for patients with a history of MI fell under four primary categories: (1) *technology functions*, (2) *social*, (3) *user needs*, and (4) *user characteristics* (n=133)*.* HP discussions fell under four different categories: (1) *technology functions*, (2) *HP involvement and research*, (3) *technology format*, and (4) *user characteristics* (n=65)*.* These were sorted from most commonly to least commonly mentioned. Feedback from round 1 (consumer needs) informed the discussions planned for the subsequent round 2 (desired functionalities). The subcategories for each round are listed under each category (Figure 2). A detailed breakdown of the advice throughout each round is provided in Multimedia Appendix 2.

#### Patients With a History of MI Advice From Round 1

Patients with a history of MI discussed *technology functions* most extensively concerning artificial intelligence, notifications, personalization, and rewards (n=47). Patients with a history of MI mentioned that they would trust the general advice provided by artificial intelligence but would check specific advice with an HP:

Does this tablet interact with something else? Asking a chat bot would be fine because they would know...if it’s something a little bit more to do with you personally I think you’d want to rely on a health professional rather than chatbots.

patient with a history of MI 1, interview

Notifications were recommended primarily for medication and should be personalized according to users’ clinical characteristics, such as time since the MI, and users’ performance on the app, such as the rate of goal achievement. Points should be used in a rewards system, and patients with a history of MI explained how social competition and fundraisers can increase intrinsic satisfaction:

It gives me little stars and rewards...you could get money or raise money or anything like that, but it was displayed on your page I guess for anyone who was sponsoring you to see.

patient with a history of MI 2, interview

Patients with a history of MI discussed *social elements* regarding family, friends and the community, and HP care (n=30). They expressed a need for support groups and peer support, which are currently unmet: “You could find people in your area to go on a walk or you could find a local sports club” (patient with a history of MI 3, interview)*.* Patients with a history of MI would use an interface with no HP interaction and would like the option to set reminders about what to ask their HP on their next visit. When asked if they would use a technology without HP interaction, 1 participant said, “You know, that would be good,” and later, “when you go in there you just blank out, you know when you go and see a medical professional” (patient with a history of MI 4, interview).

Patients with a history of MI also discussed *user needs*, including intervention modality, barriers, education, and tracking (n=30). Most patients with a history of MI preferred a simple app modality over other modalities because of its convenience. Barriers included the user’s technical ability to set up and use an app, containing relevant information for individual users, and the user’s motivation:

It was just so complicated. I just didn’t have the strength or the ability to go out and try and work out how to use an Apple Watch and then coordinate it to my heartbeat and then send it to him. So that was all in the too-hard basket.

patient with a history of MI 4, interview

Education on the effects of surgeries and medication, risk factors, exercise, cardiopulmonary resuscitation, and diet guidelines and the next steps in the users’ journey were identified as a facilitator for app use: “Sodium and stuff like that, what are good, what are levels sort of OK to have?” (patient with a history of MI 2, interview). Tracking was highly emphasized, including sleep, pain symptoms, medication, diet, exercise, smoking, pulse, oxygen levels, steps, calories, blood pressure, cardiac blues and mood, and weight.

Finally, patients with a history of MI advised on *user characteristics*, including the age of users, state of mind, and statistics of a future MI (n=26). Patients with a history of MI advised that some older adults would not have an issue with technology use and that experiencing an MI is often motivation enough to change negative lifestyle behavior:

It’s really hard in terms of like motivating, without having had...that big scare.

patient with a history of MI 2, interview

Everything is going is all digital...look at it more rather than if you go and gave them a pamphlet.

patient with a history of MI 1, interview

Patients with a history of MI were keen to receive statistics concerning the risk of recurrent MI episodes to stimulate behavior change. They would also welcome prompts that emphasize the need for behavior change in addition to surgical and medication treatments. Patients with a history of MI emphasized extreme depression, anxiety, and loneliness after their MI:

A lot of anxiety that people may have never had, especially when you learn to be on your own after a heart attack and you’ve got to be prepared if you have another one...the people who are alone might need that extra support.

patient with a history of MI 4, interview

#### HP Advice From Round 1

HPs discussed *technology functions* most extensively regarding the use of artificial intelligence; rewards; personalization; tracking; and provision of education and support from family, friends, and the community (n=36). Some HPs were unsure whether patients with a history of MI would understand artificial intelligence: “I think 50/50 [would use artificial intelligence]...I think we give the person the choice” (HP 1, interview). They suggested catering to education levels by providing relatively easy content to understand. HPs suggested that a family member of a patient with a history of MI can be made into a “champion” over the app, where they can keep track of the user’s health goals. Patients with a history of MI can have a “champion in their app that they connect the patient to one of your family or friends and they get like a very good message, supportive and motivational from their supporters, from their champion. This is one of the ways that we can like reinforce their behaviour change as well” (HP 2, FGD). An incentives program would motivate patients with a history of MI with rewards, including a fruit hamper or a personal training session. The app should be personalized based on time since MI and specific user goals:

The most important element is time. What sort of information or education the person needs in the first 24-48 hours after discharge...[where] we do expect recurrence of the heart attack much more than what would happen in the months after.

HP 3, FGD

HPs mentioned that tracking capabilities should include a digital Webster pack and daily diary.

*HP involvement and research* were discussed, including communication with an HP and using a theory-based approach (n=11). They emphasized the importance of the output being clinically sound and interpretable by HPs: “It should be clinically sound and logical, so the information they get should be meaningful and interpretable by a healthcare professional” (HP 3, FGD). They also suggested that theoretical approaches are necessary, such as the “diffusion of innovation theory” (HP 3, FGD), which tailors different approaches to patient technology adoption behaviors [33]:

We can incorporate different theories, behaviour change theories. And we need to start involving and engaging patient in the design.

HP 2, FGD

HPs emphasized an appropriate *technology format*, including a feedback mechanism, making it fun and simple (n=9). HPs encouraged feedback loops based on user behavior to improve motivation. This feedback loop “gives them hope and it gives them an understanding as to how they are they’re doing” (HP 3, FGD). Simple formatting with a hierarchical structure that incorporates visuals and games, such as a visual of a tree thriving when app use is high or suffering if app use is low, would make the app appealing:

That application about time management...when you know you’re not able to finish that [study] time of 45 minutes that you already plan, it’s like someone is cutting a tree, that it makes you somehow guilty.

HP 4, FGD

Finally, certain *user characteristics* should be considered in app development, such as socioeconomic demographics, older adult population, comorbidities, and users’ state of mind (n=9). To make the app affordable for all socioeconomic groups, developers should consider whether subscriptions or one-time purchases are appropriate. Some older adults would need to be guided on how to download an app. HPs stated that health needs should be determined based on each user’s comorbidities, and self-care, uncertainty, and mental health symptoms such as health-related stress should be supported. It is common that patients with a history of MI will “not know what to do about the condition. Frightened to ask for more care. Lacking confidence and continuing to be independent” (HP 1, interview).

### Round 2: Desired Functionalities

#### Overview

The advice from round 1 (consumer needs) was discussed with patients with a history of MI and HPs concerning round 2 (desired functionalities). In round 2, we walked participants through a conceptual design and asked for specific advice about features that were emphasized in round 1, such as social support. Example screenshots of this design are available in Multimedia Appendix 3. The desired functionalities were determined from the previous round to be unmet needs or opportunities. We organized the discussion points from the desired functionalities round into four primary categories: (1) *social*, (2) *app development*, (3) *mental health*, and (4) *artificial intelligence.* These were sorted from most to least commonly discussed. These categories were applicable to both patients with a history of MI (n=230) and HPs (n=256) because the targeted line of questioning was designed to elicit specific and detailed responses.

#### Patients With a History of MI Advice From Round 2

Patients with a history of MI detailed *social* support features, including the creation of a “champion,” discussion forum, success stories, and support groups (n=76). Patients with a history of MI preferred the term “buddy” to champion, stating that a buddy should have experienced an MI and can communicate with the user over the app:

Somebody who’s actually had the experience, because my wife’s holding me accountable. She doesn’t understand how I feel half the time.

patient with a history of MI 5, FGD

An artificial intelligence buddy was suggested for users who wanted only information and reminders rather than a personal connection: “If you want total objective-type support then an automated agent would be really good” (patient with a history of MI 5, FGD). Patients with a history of MI had a high interest in the discussion forum, suggesting filtering discussions based on conditions and receiving personalized notifications about liked threads. They mentioned the importance of short success stories and support groups with consistent scheduling systems. Patients with a history of MI preferred the support groups to be structured with presentations from HPs, followed by a patients with a history of MI–led question and answer session:

I would prefer the presentation and then people can join in and ask questions...that will really get people talking.

patient with a history of MI 2, interview

Patients with a history of MI discussed *app development*, including additional features, development, app features, and collaboration (n=57). Additional features include explicit references to personalization capability, links to social media, journaling functions, and further tracking features, including blood pressure, oxygen, subjective pain, and other sicknesses. Patients with a history of MI expressed the need for developers to store large files on a server or YouTube to reduce phone storage requirements: “Make the app not chunky with megabytes...there’s premium space on a lot of phones” (patient with a history of MI 6, FGD). Features included downloading a report to show their HP and GPS tracking for the user to avoid their personal, habitual smoking or alcohol locations. One patient with a history of MI said the GPS tracking can inform users to “‘Quit, turn around. Go the other way. Avoid at all costs.’ Probably a warning of some form could pop up” (patient with a history of MI 7, interview). Finally, collaborating with a corporate partner would facilitate app development and rollout.

Patients with a history of MI spoke about the importance of *mental health*, including additional features, lived experience, mental health states, and tools (n=51). Additions include fun exercises that can raise mood, progressive muscle relaxation to improve sleep, and encouraging hobbies (eg, gardening): “‘Have you done your exercises today?’ just because you know, that releases the endorphins or something and makes you feel good” (patient with a history of MI 7, interview)*.* Patients with a history of MI stated that isolation is particularly anxiety-inducing because of the looming fear of death. Along with anxiety, patients with a history of MI mentioned other mental states that should be addressed, including mood, depression, social uncertainty (eg, loved ones coping with their condition), sleep, maintaining positivity, mortality, and memory loss: “A lot of it’s the focus on how do you cope with your condition? But we have to think about the people we interact with, how they cope with the condition?” (patient with a history of MI 5, FGD). Mental health modules should address these issues and can be organized based on the lived experience. Mindfulness exercises should emphasize the perception of the body and heartbeat so that patients with a history of MI can notice heartbeat abnormalities. Mindfulness of the body can help patients with a history of MI to “go and get help earlier. Probably a simple equation, you know something’s not right and get it fixed. Get it looked at and in doing that, that’s what saved my heart muscle” (patient with a history of MI 8, interview).

Finally, *artificial intelligence* was discussed, including behavior patterns and a chatbot (n=46). Visualizing user patterns of behavior can motivate improvement, with diet, exercise, mental health, smoking, and drinking identified as the most important to visualize. Patients with a history of MI are willing to use the chatbot, particularly for subjectively uncomfortable or embarrassing questions that they would not ask an HP. For example, asking “a question you think is a really dumb question, or you’re not sure. You can start with the chatbot and you get a response and you don’t feel like you’re embarrassing yourself because it’s just a machine” (patient with a history of MI 5, FGD). The chatbot should use links and information from trusted sources and should mostly answer general questions, “as long as they’re not sending me links to Wikipedia” (patient with a history of MI 9, interview). Patients with a history of MI stated that it should be adaptive, for example, if a low mood is recorded, it should suggest an intervention for low mood. However, patients with a history of MI would seek an HP’s opinion regardless of the chatbot, and there is a concern that the chatbot may not understand the user’s questions: “If I get stuck in a vicious cycle, the chatbot should somehow be configured to understand that it’s caught in a loop...then issue an alternative resource link or number” (patient with a history of MI 10, FGD).

#### HP Advice From Round 2

HPs detailed *social* support, including additional features, a “champion,” a discussion forum and a support group (n=95). Additions include outdoor activities such as park runs and promoting healthy restaurants to try with friends. HPs labeled the “champion” as a “buddy” who should undergo training, have a positive attitude toward change, and be matched to the user based on needs. This can include the “opportunity to have a little bit of training about motivational interviewing, etc. Like, you don’t want someone saying, ‘Oh, you know, that’s hopeless. How come you started smoking again?’...maybe they’d have access to a ‘be a champion app,’ which would give them hints what to do when someone’s not achieved a goal” (HP 5, FGD). They suggested that users can find a buddy from the support groups or discussion forums rather than appointing a family member (to reduce familial conflict) or use the chatbot as their buddy. They emphasized the need for a disclaimer on the discussion forum for potential misinformation and the ability to flag an item to be removed. The support groups should be a combination of (1) an open forum to share experiences and (2) presentations from an HP with a question-and-answer session. There should be group polls to choose discussion topics and a calendar for these recorded sessions running with an HP moderator: “Ahead of [the] week, we can ask them couple of questions and ask them which topic they prefer to talk in the next session” (HP 2, interview). The app should include an introductory recorded session to encourage users to participate.

HPs discussed *app development* including additional features or changes, legalities, other organizations, and the use of app features (n=66). Additions include a simple daily plan, blood pressure tracking, a monthly report, and a road map for the future:

Having that long-term vision and knowing that they’re on the right pathway and they’re doing the right things can be really beneficial. I’m all for having a really clear plan.

HP 6, FGD

HPs advised on further visual appeals, such as larger headings and videos. HPs emphasized privacy regulations and consent if support groups are recorded and suggested involving cardiac rehabilitation staff for advertisements:

People aren’t going to stumble across it. Yeah, and maybe you should think about getting it as endorsed as a product through ACRA or Heart Foundation or linking with some of those organisations so that people are actually channelled there.

HP 6, FGD

GPS tracking to avoid the habitual smoking or drinking locations of patients with a history of MI is very novel and could be useful with large warnings to alert the user when nearing these locations.

HPs discussed *artificial intelligence*, including behavior patterns and a chatbot (n=49). Artificial intelligence should not be labeled or obvious to users. Personalized behavior patterns are likely to be used and should report on overall health, for example, patterns for chest pain symptoms and types of exercise. HPs suggested that the chatbot should both (1) enable specialist appointment booking and (2) answer both general and specific questions tailored to the user. The addition of an avatar can make the chatbot more personable: “Have an avatar and make it look like a person” (HP 7, interview). Overall, many patients with a history of MI will not completely trust the chatbot but may use it:

To me a chatbot is another tool in a large toolbox, so people may not use it. You don’t need a screwdriver every single day. But you may need a screwdriver one day, and if you know how a screwdriver works, then you will refer back to it.

HP 3, interview

Finally, HPs discussed *mental health*, including mental health states, support from family members, tools, and usefulness (n=46). Patients with a history of MI can struggle with mental health more than with physical health:

Being in cardiac rehab for the many years...this is the biggest part of it. Now, it’s going to always be more mental than physical.

HP 8, FGD

The feelings of patients with a history of MI of being broken, uncertain, and alone, along with the effect of quitting smoking and a changed sex life, should be addressed. HPs stated the app should support family members, who may also experience declined mental health, with dedicated modules (potentially in a “family member” app or section of the app):

Cover family emotions as well...because there’s often been a bigger event for the family than it has been for the client, which sounds funny, but it’s because they obviously don’t remember any of it. Whereas, the family, say a wife who’s witnessed it, or maybe done CPR and stuff like that, it’s been a really horrific event for her.

HP 9, interview

Mental health modules should have problem-focused outcomes based on the experiences of patients with a history of MI. HPs stated that mindfulness exercises should be tailored to mental health states, for example, different audio recordings for depression and anxiety. Overall, mental health was highly regarded by the HPs.

### Round 3: Simulation-Informed Feedback

#### Overview

The advice provided from round 2 (desired functionalities) was extrapolated into a simulated design that was critiqued in round 3 (simulation-informed feedback). In round 3, we showed participants a mock-up prototype that they could interact with and asked for advice about features that apply to the full application, such as engagement features. The information derived from round 3 is presented in the following sections. Results from the patients with a history of MI were displayed in four primary categories: (1) *barriers and solutions*, (2) *app use*, (3) *engagement*, and (4) *suggestions* (n=286). Whereas HP discussions fell under three categories: (1) *development and rollout*, (2) *novelty effect*, and (3) *concerns* (n=96). Feedback from round 3 is listed in subsequent sections, sorted from most to least commonly discussed.

#### Patients With a History of MI Advice From Round 3

Patients with a history of MI discussed *barriers and solutions to these barriers*, including complexity, privacy, encouraging app use, and motivation (n=112). Patients with a history of MI suggested gradually introducing new features based on progress, for example, “Somebody who started off perhaps with the simple concept, once they got the hang of it and became familiar with it and comfortable, would then step up to the next level” (patient with a history of MI 11, FGD). Users should be able to choose a specific page that automatically loads every morning. Patients with a history of MI mentioned that upon setup, the app should ascertain features that users would use and only display these features. Push notifications can be used especially during extensive idle periods, for example, “It’s been a month since you last did a health check. Would you like to go to the to the quick check page?” (patient with a history of MI 12, FGD). Organizational health sponsors can provide patients with a history of MI with a percentage of their product if goals are achieved.

Patients with a history of MI also discussed *app use*, including its features and functions (n=101). Many web-based resources are based on standards from other countries, for example, the United States; Australian standards and education are required: “It’d be good to have an Aussie one because the nutritional information is quite different” than American information (patient with a history of MI 9, FGD). Patients with a history of MI mentioned that their family members and carers should have their own app log-in to receive relevant support and education. Correlational graphs could show the link between biometric health data and activity levels or medication use:

So just being able to add different things into this section is good as well...then it shows you know, if you’ve missed a tablet, the effect of missing that tablet is your blood pressure goes up.

patient with a history of MI 13, FGD

Patients with a history of MI spoke about increasing *engagement*, including competitions, gamification, and rewards (n=51). Patients with a history of MI were unsure if they would compete with a large group. Rather, they would play a game or a competition against themselves or a buddy. The app should provide participation achievements rather than specific outcomes (eg, step count) because “everybody’s different in their ability, in what they’d be able to do” (patient with a history of MI 15, FGD). Overall goal achievement across the app can mitigate negative self-talk, for example, “62% of users reach their food goals, gives you an indication that that’s okay. It’s literally only just over half. Therefore, the fact that I didn’t reach my food goals is probably not that big a deal. But by the same token, you know, hey, it’d be nice to be in that 62%” (patient with a history of MI 14, interview). Patients with a history of MI suggests that with consistent app use, the user can earn a gamified level, such as “guru,” displayed on the discussion forum. Rewards can include obtaining a percentage of certain brands, entry to a relevant health conference, or a one-on-one with the app’s HP:

Medals are good, but they tend to have the lifespan is not great...in the long run, it’s just an achievement on an app...especially if you’re dangling like bigger, will say the carrot or fruit in front of it, you can have a one-on-one with a cardiologist...which is very, very valuable even the private system and in the public system.

patient with a history of MI 13, FGD

Concern that users would cheat led to a discussion on nontangible rewards. Nonmonetary rewards could involve expressive emojis, and medals were perceived as childish.

Patients with a history of MI provided additional *suggestions*, including added content to the app and technical changes (n=22). Content should include other contributors that affect physical symptoms (eg, the effect of weather on the heart): “It’s the inflammation [caused by] I think the variations in climate that we get from all in one day” (patient with a history of MI 16, FGD). Patients with a history of MI suggested including a page for resources and emergency contacts for each user. The app should be relatively small and enabled to synchronize with the cloud, the phone’s calendar system, family members’ calendars, the Apple or Samsung Health app, Bluetooth devices, and other fitness apps. The app should allow the recording of heart rates for enabled phones.

#### HP Advice From Round 3

HPs discussed the *development and rollout of the app*, including the involvement of HPs, the likelihood of app use, and technical app development (n=39). An HP should be able to communicate with patients with a history of MI through the app for a check-up:

You know, building in those specialist follow ups, and maybe that would be something that when you talk about the journey, that that may be something that we could build into that journey as well, because it is able to be tailored and customized.

HP 5, FGD

The app should be introduced during or near the end of standard cardiac rehabilitation and framed as ongoing support. The app can “be that sort of bridge for when people do finish [cardiac rehabilitation], that they’ve got somewhere else to go and you know, some of the options available to them” (HP 10, FGD). HPs stated that patients with a history of MI can meet potential app buddies with others involved in cardiac rehabilitation, creating a personal connection. Notifications should decrease in frequency over time and be titrated to determine how often the patients with a history of MI complete their goals.

HPs discussed ways to overcome the *novelty effect*, including fostering engagement and integrating the app into the user’s lifestyle (n=33). Competitions without associated disappointment can foster engagement with the app, but if the leaderboard becomes demotivating, it can be removed:

My concern is for those people that are lower down on the leaderboard each time. Is that going to be something where they start to go, “oh, well, I’m not achieving like those people” and set them up for that disappointment.

HP 5, FGD

The app should have the functionality to see friends’ activity levels to motivate the activity levels of patients with a history of MI. Points can tie in with a heart-healthy sponsor for discounts, but HPs are unsure whether patients with a history of MI require this external reward for effective behavior change. Upon installation, videos of a navigator should be used to guide new users through activities and modules. Patients with a history of MI “wanted somebody to guide them through what each stage meant, and what they would get out of it. So that they went into each section of [a] module and, perhaps the same for this, knowing what they expect to get out of it” (HP 11, FGD). This navigator should be a “patient. It’s actually a consumer, consumer who’s guiding people through” (HP 11, FGD). HPs suggested integrating goals on an adaptive figure of a heart that changes based on goal progress, for example, a healthy heart is displayed if goals are achieved.

Finally, HPs discussed *concerns*, including interest, motivation, superfluous features, and the transition to technology (n=24). They suggested that a dedicated health care team presenting content not otherwise available would maintain the interest of patients with a history of MI in the app: “Are you actually going to be doing your own videos?...I think it’d be great if, yeah, as much as possible” (HP 11, FGD). HPs stated that no features or functions are superfluous, although reducing manually entered information, visual clutter, and redundant notifications could maintain the interest of patients with a history of MI:

No, I don’t think it’s overwhelming...I think it’s better to have more than less, I think sometimes you go on apps, and you think I’d just like to have this but it’s just not available

HP 10, FGD

A companion website could simplify manual data entry, but the transition to technology will depend on each user with variability in affinity with technology. One participant asked if “you have to do it all on your phone, like you can’t go online and enter stuff in?...I’m just thinking practically, like, you’ve got people [that] don’t want to be typing on their phone” (HP 6, FGD).

## Discussion

### Principal Findings

This study aimed to specify the key needs identified by patients with a history of MI and HPs for the secondary prevention of MI using digital health technology. The results from the 3 rounds of FGDs provide rich information about the various primary needs of end users, the methods and structures on how to provide these needs in digital health technology, and the practicalities of rolling out the app to patients with a history of MI. We will organize the discussion of findings according to two main themes: (1) topics that patients with a history of MI and HPs agreed on and (2) topics on which patients with a history of MI and HPs had differing opinions. For theme 1, these topics included addressing mental health; the fit of the intervention within current health care (ie, the timing of app disbursement); and features of the app, including a focus on family members and engagement. For theme 2, these topics included a lack of social support and the use of artificial intelligence to deliver support. We start by discussing theme 1 in the context of prior research.

### Comparison With Prior Work

Research emphasizes the impact of MI on the exacerbation of mental health problems and that decreased mental health can lead to poorer heart health outcomes [34,35]. However, our participants stated that there is a lack of support for mental health and a strong desire for greater support. Participants emphasized specific areas of mental health, including normalizing feelings of depression, anxiety, loneliness and not feeling “whole;” providing education to patients with a history of MI, such as awareness of warning signs for mental health issues; tools to assess and track mental health; intervention techniques, such as mental health modules and mindfulness activities; and using social formats, such as discussion forums. These needs have also been stressed in the mental health literature [36] but not incorporated into cardiology-related interventions. Therefore, our findings provide specific approaches for improving mental health in patients with a history of MI by using digital health tools.

Both patients with a history of MI and HPs agreed that incorporating the app during or immediately after cardiac rehabilitation would ensure the best likelihood of habit formation and long-term use of the app. A similar app provided directly after cardiac rehabilitation was able to demonstrate this habit maintenance and low attrition (n=2) after a 1-year follow-up, with improved peak oxygen uptake, exercise performance and habits, and self-perceived goal achievement in comparison with a control [37]. Participants in this study mentioned that users may consider the app to be an extension of cardiac rehabilitation, contributing to their earnest and consistent long-term use of the app.

Both patients with a history of MI and HPs agreed that family members of patients with a history of MI are often neglected in postdischarge care. By incorporating app modules or a version of the app designed for family members with a focus on promoting education and mental health for these family members, they will be best able to provide frequent support to patients with a history of MI in their recovery journey. This need is reflected in previous research, in which cardiac rehabilitation educational programs for patients and family members have been theoretically developed [38]. The involvement of family members in each stage of rehabilitation was found to result in improved exercise tolerance, quality of life, perceived stress, and state anxiety [39]. Therefore, there is merit in involving family members in the care of patients with a history of MI that can potentially alleviate poor reported mental health symptoms.

Finally, strategies to increase engagement with digital health were similarly discussed between patients with a history of MI and HPs. There is limited research addressing engagement in the MI literature, but research on engagement (eg, leader boards and points systems) to increase physical activity has shown promise [40,41]. Patients with a history of MI and HPs advised the use of a simple game with a points system that levels up the user’s profile, which is displayed on the discussion forum. They stated that points can also be earned together with a buddy when both users achieve their goals, aimed at eliciting a social responsibility to achieve goals [41]. Competitions were viewed less favorably, with both patients with a history of MI and HPs stating that it can easily become demotivating and can potentially be omitted. Therefore, implementing engaging games rather than competitions is a well-valued strategy for improving long-term engagement. We now discuss the contrasts in advice between patients with a history of MI and HPs.

Patients with a history of MI emphasized the lack of support from their health care team and a lack of understanding from their family and community more strongly than HPs. Negative health outcomes are exacerbated in patients with a history of MI without a support network [42,43]. However, many participants reported no awareness of support groups, despite their desire to attend a support group. Participants agreed with previous research that implementing a real-time support group using videoconferencing can maintain patient involvement [44]. Both patients with a history of MI and HPs stated that introducing a buddy system will provide patients with a history of MI with a real connection to combat feeling alone in their disorder. However, HPs particularly emphasized that the buddy should not be a personal relationship of the patients with a history of MI, whereas the patients with a history of MI stated that the buddy should be anyone who has also experienced an MI. Therefore, social support as a critical facet of postdischarge care is highly significant for the population of patients with a history of MI, who strongly emphasized this unmet need.

Artificial intelligence is increasingly used in cardiology research [45]. Patients with a history of MI expressed some openness to intentionally experimenting with artificial intelligence, whereas HPs stated that patients with a history of MI should not know that artificial intelligence is being used, expressing it would work more efficiently in the back end of the app. Artificial intelligence chatbots have been shown to have high efficacy in promoting health behavior change among diverse populations, including promoting healthy lifestyles, smoking cessation, or treatment or medication adherence and reducing substance misuse but with poor feasibility, usability, and acceptability [46]. Therefore, co-designed approaches, as in this study, may be needed to develop an acceptable chatbot for patients with a history of MI. Patients with a history of MI particularly emphasized the importance of the chatbot appearing on the side of the app screen at relevant and opportune times. For example, after tracking low mood, the chatbot can appear and suggest modules to support mood. This builds on previous research findings that chatbots should provide real-time reinforcement and on-demand support [46]. Finally, HPs expected personalized responses from the chatbot. However, patients with a history of MI stated that general information is more trustworthy because it is less likely to be affected by chatbot interpretation and that general responses are preferred compared with having no chatbot functionality.

### Strengths and Limitations

A strength of this study was its iterative approach, with key needs being refined and confirmed with both participant groups multiple times. Participants involved in subsequent rounds were either the same participants from the previous rounds—where their comments could be clarified using a visual design—or new participants, where new advice could be provided. Therefore, the information derived from the rounds was rich with new insights and feedback provided in each round.

Participants were relatively young for an MI target population. The mean age of patients with a history of MI across all rounds was 47 (SD 12.13) years, whereas the average age at the first MI was 65.2 years [47]. The web-based advertisements and videoconference format likely led those who are more technologically literate, perhaps younger, to express an interest in the study. Therefore, the results should be interpreted based on the age range of the participants. The number of participants in each focus group (maximum=5) was relatively small compared with the reported median of 10 participants [48]. This may limit discussion between many participants but allowed us to overcome the issue of fragmented communication inherent with web-based videoconferencing. Finally, round 1 only contained advice from 8 participants. However, considering that all rounds included 38 participants, the study included an adequate number of participants compared with other studies [28].

### Conclusions

We gathered insights from patients with a history of MI and HPs regarding the need for a digital health solution for the secondary prevention of MI. Both patients with a history of MI and HPs highlighted focusing on mental health, collaborating with heart health organizations, involving family members in postdischarge care, and increasing engagement in simple games. These results can inform the development of a valued digital health secondary prevention strategy for patients with a history of MI. Future research should conduct a pilot study using the findings of the MiSmartHeart study to guide intervention development.

## Appendix

Multimedia Appendix 1 Full focus group and interview question guide.

Multimedia Appendix 2 Advice provided in each round.

Multimedia Appendix 3 Example screenshots of conceptual and simulated designs.

## Abbreviations

FGD: focus group discussion
HP: health professional
mHealth: mobile health
MI: myocardial infarction

## Data Availability

The data sets generated during and analyzed during this study are not publicly available because of the sensitive and personal information divulged in the discussions but are available from the corresponding author upon reasonable request.

## References

[1] Johansson S, Rosengren A, Young K, Jennings E (2017). Mortality and morbidity trends after the first year in survivors of acute myocardial infarction: a systematic review. BMC Cardiovasc Disord.

[2] Dunlay SM, Pack QR, Thomas RJ, Killian JM, Roger VL (2014). Participation in cardiac rehabilitation, readmissions, and death after acute myocardial infarction. Am J Med.

[3] Martin BJ, Arena R, Haykowsky M, Hauer T, Austford LD, Knudtson M, Aggarwal S, Stone JA (2013). Cardiovascular fitness and mortality after contemporary cardiac rehabilitation. Mayo Clin Proc.

[4] Herber OR, Smith K, White M, Jones MC (2017). 'Just not for me' - contributing factors to nonattendance/noncompletion at phase III cardiac rehabilitation in acute coronary syndrome patients: a qualitative enquiry. J Clin Nurs.

[5] Clark AM, King-Shier KM, Spaling MA, Duncan AS, Stone JA, Jaglal SB, Thompson DR, Angus JE (2013). Factors influencing participation in cardiac rehabilitation programmes after referral and initial attendance: qualitative systematic review and meta-synthesis. Clin Rehabil.

[6] Rouleau CR, King-Shier KM, Tomfohr-Madsen LM, Aggarwal SG, Arena R, Campbell TS (2018). A qualitative study exploring factors that influence enrollment in outpatient cardiac rehabilitation. Disabil Rehabil.

[7] Akesson A, Larsson SC, Discacciati A, Wolk A (2014). Low-risk diet and lifestyle habits in the primary prevention of myocardial infarction in men: a population-based prospective cohort study. J Am Coll Cardiol.

[8] Teo K, Lear S, Islam S, Mony P, Dehghan M, Li W, Rosengren A, Lopez-Jaramillo P, Diaz R, Oliveira G, Miskan M, Rangarajan S, Iqbal R, Ilow R, Puone T, Bahonar A, Gulec S, Darwish EA, Lanas F, Vijaykumar K, Rahman O, Chifamba J, Hou Y, Li N, Yusuf S (2013). Prevalence of a healthy lifestyle among individuals with cardiovascular disease in high-, middle- and low-income countries: the Prospective Urban Rural Epidemiology (PURE) study. JAMA.

[9] Iyengar K, Upadhyaya GK, Vaishya R, Jain V (2020). COVID-19 and applications of smartphone technology in the current pandemic. Diabetes Metab Syndr.

[10] Jiang X, Ming WK, You JH (2019). The cost-effectiveness of digital health interventions on the management of cardiovascular diseases: systematic review. J Med Internet Res.

[11] Burke LE, Ma J, Azar KM, Bennett GG, Peterson ED, Zheng Y, Riley W, Stephens J, Shah SH, Suffoletto B, Turan TN, Spring B, Steinberger J, Quinn CC (2015). Current science on consumer use of mobile health for cardiovascular disease prevention: a scientific statement from the American heart association. Circulation.

[12] Widmer RJ, Collins NM, Collins CS, West CP, Lerman LO, Lerman A (2015). Digital health interventions for the prevention of cardiovascular disease: a systematic review and meta-analysis. Mayo Clin Proc.

[13] Şaylık F, Çınar T, Hayıroğlu Mİ, Tekkeşin Aİ (2023). Digital health interventions in patient management following acute coronary syndrome: a meta-analysis of the literature. Anatol J Cardiol.

[14] Leong DP, Joseph PG, McKee M, Anand SS, Teo KK, Schwalm JD, Yusuf S (2017). Reducing the global burden of cardiovascular disease, part 2: prevention and treatment of cardiovascular disease. Circ Res.

[15] Tekkeşin Aİ, Hayıroğlu Mİ, Çinier G, Özdemir YS, İnan D, Yüksel G, Pay L, Parsova KE, Vatanoğlu EG, Şeker M, Durak F, Gürkan K (2021). Lifestyle intervention using mobile technology and smart devices in patients with high cardiovascular risk: a pragmatic randomised clinical trial. Atherosclerosis.

[16] Johnston N, Bodegard J, Jerström S, Åkesson J, Brorsson H, Alfredsson J, Albertsson PA, Karlsson J, Varenhorst C (2016). Effects of interactive patient smartphone support app on drug adherence and lifestyle changes in myocardial infarction patients: a randomized study. Am Heart J.

[17] Chow CK, Redfern J, Hillis GS, Thakkar J, Santo K, Hackett ML, Jan S, Graves N, de Keizer L, Barry T, Bompoint S, Stepien S, Whittaker R, Rodgers A, Thiagalingam A (2015). Effect of lifestyle-focused text messaging on risk factor modification in patients with coronary heart disease: a randomized clinical trial. JAMA.

[18] Shariful Islam SM, Farmer AJ, Bobrow K, Maddison R, Whittaker R, Pfaeffli Dale LA, Lechner A, Lear S, Eapen Z, Niessen LW, Santo K, Stepien S, Redfern J, Rodgers A, Chow CK (2019). Mobile phone text-messaging interventions aimed to prevent cardiovascular diseases (Text2PreventCVD): systematic review and individual patient data meta-analysis. Open Heart.

[19] Wald DS, Bestwick JP, Raiman L, Brendell R, Wald NJ (2014). Randomised trial of text messaging on adherence to cardiovascular preventive treatment (INTERACT trial). PLoS One.

[20] Coorey GM, Neubeck L, Mulley J, Redfern J (2018). Effectiveness, acceptability and usefulness of mobile applications for cardiovascular disease self-management: systematic review with meta-synthesis of quantitative and qualitative data. Eur J Prev Cardiol.

[21] Palacholla RS, Fischer N, Coleman A, Agboola S, Kirley K, Felsted J, Katz C, Lloyd S, Jethwani K (2019). Provider- and patient-related barriers to and facilitators of digital health technology adoption for hypertension management: scoping review. JMIR Cardio.

[22] Slevin P, Kessie T, Cullen J, Butler MW, Donnelly SC, Caulfield B (2020). Exploring the barriers and facilitators for the use of digital health technologies for the management of COPD: a qualitative study of clinician perceptions. QJM.

[23] Roth JA, Battegay M, Juchler F, Vogt JE, Widmer AF (2018). Introduction to machine learning in digital healthcare epidemiology. Infect Control Hosp Epidemiol.

[24] Istepanian RS, Al-Anzi T (2018). m-Health 2.0: new perspectives on mobile health, machine learning and big data analytics. Methods.

[25] Papoutsi C, Wherton J, Shaw S, Morrison C, Greenhalgh T (2021). Putting the social back into sociotechnical: case studies of co-design in digital health. J Am Med Inform Assoc.

[26] Steen M, Manschot M, de Koning N (2011). Benefits of co-design in service design projects. Int J Des.

[27] Noorbergen TJ, Adam MT, Roxburgh M, Teubner T (2021). Co-design in mHealth systems development: insights from a systematic literature review. AIS Trans Comput Hum Interact.

[28] Eyles H, Jull A, Dobson R, Firestone R, Whittaker R, Te Morenga L, Goodwin D, Mhurchu CN (2016). Co-design of mHealth delivered interventions: a systematic review to assess key methods and processes. Curr Nutr Rep.

[29] Pereira RB, Brown TL, Guida A, Hyett N, Nolan M, Oppedisano L, Riley K, Walker G (2021). Consumer experiences of care coordination for people living with chronic conditions and other complex needs: an inclusive and co-produced research study. Aust Health Rev.

[30] Schemer L, Hess CW, Van Orden AR, Birnie KA, Harrison LE, Glombiewski JA, Simons LE (2023). Enhancing exposure treatment for youths with chronic pain: co-design and qualitative approach. J Particip Med.

[31] Kim H, Sefcik JS, Bradway C (2017). Characteristics of qualitative descriptive studies: a systematic review. Res Nurs Health.

[32] Sandelowski M (2000). Whatever happened to qualitative description?. Res Nurs Health.

[33] Dearing JW, Cox JG (2018). Diffusion of innovations theory, principles, and practice. Health Aff (Millwood).

[34] Nielsen TJ, Vestergaard M, Christensen B, Christensen KS, Larsen KK (2013). Mental health status and risk of new cardiovascular events or death in patients with myocardial infarction: a population-based cohort study. BMJ Open.

[35] Rutledge T, Redwine LS, Linke SE, Mills PJ (2013). A meta-analysis of mental health treatments and cardiac rehabilitation for improving clinical outcomes and depression among patients with coronary heart disease. Psychosom Med.

[36] Lal S, Adair CE (2014). E-mental health: a rapid review of the literature. Psychiatr Serv.

[37] Lunde P, Bye A, Bergland A, Grimsmo J, Jarstad E, Nilsson BB (2020). Long-term follow-up with a smartphone application improves exercise capacity post cardiac rehabilitation: a randomized controlled trial. Eur J Prev Cardiol.

[38] de Melo Ghisi GL, Scane K, Sandison N, Maksymiu S, Skeffington V, Oh P (2015). Development of an educational curriculum for cardiac rehabilitation patients and their families. J Clin Exp Cardiol.

[39] Vahedian-Azimi A, Miller AC, Hajiesmaieli M, Kangasniemi M, Alhani F, Jelvehmoghaddam H, Fathi M, Farzanegan B, Ardehali SH, Hatamian S, Gahremani M, Mosavinasab SM, Rostami Z, Madani SJ, Izadi M (2016). Cardiac rehabilitation using the Family-Centered Empowerment Model versus home-based cardiac rehabilitation in patients with myocardial infarction: a randomised controlled trial. Open Heart.

[40] Ganesan AN, Louise J, Horsfall M, Bilsborough SA, Hendriks J, McGavigan AD, Selvanayagam JB, Chew DP (2016). International mobile-health intervention on physical activity, sitting, and weight: the Stepathlon Cardiovascular Health Study. J Am Coll Cardiol.

[41] Patel MS, Benjamin EJ, Volpp KG, Fox CS, Small DS, Massaro JM, Lee JJ, Hilbert V, Valentino M, Taylor DH, Manders ES, Mutalik K, Zhu J, Wang W, Murabito JM (2017). Effect of a game-based intervention designed to enhance social incentives to increase physical activity among families: the BE FIT randomized clinical trial. JAMA Intern Med.

[42] Hakulinen C, Pulkki-Råback L, Virtanen M, Jokela M, Kivimäki M, Elovainio M (2018). Social isolation and loneliness as risk factors for myocardial infarction, stroke and mortality: UK Biobank cohort study of 479 054 men and women. Heart.

[43] Compare A, Zarbo C, Manzoni GM, Castelnuovo G, Baldassari E, Bonardi A, Callus E, Romagnoni C (2013). Social support, depression, and heart disease: a ten year literature review. Front Psychol.

[44] Bashi N, Hassanzadeh H, Varnfield M, Wee Y, Walters D, Karunanithi M (2018). Multidisciplinary smartphone-based interventions to empower patients with acute coronary syndromes: qualitative study on health care providers' perspectives. JMIR Cardio.

[45] Johnson KW, Torres Soto J, Glicksberg BS, Shameer K, Miotto R, Ali M, Ashley E, Dudley JT (2018). Artificial intelligence in cardiology. J Am Coll Cardiol.

[46] Aggarwal A, Tam CC, Wu D, Li X, Qiao S (2023). Artificial intelligence-based chatbots for promoting health behavioral changes: systematic review. J Med Internet Res.

[47] Mercado-Lubo R, Yarzebski J, Lessard D, Gore J, Goldberg RJ (2020). Changing trends in the landscape of patients hospitalized with acute myocardial infarction (2001 to 2011) (from the Worcester Heart Attack Study. Am J Cardiol.

[48] Nyumba TO, Wilson K, Derrick CJ, Mukherjee N (2018). The use of focus group discussion methodology: insights from two decades of application in conservation. Methods Ecol Evol.

