# Supplementary material for: Digital Health Secondary Prevention Using Co-Design Procedures: Focus Group Study With Health Care Providers and Patients With Myocardial Infarction
Source: JMIR Cardio. 2023 Oct 30;7:e49892. doi: 10.2196/49892 (PMC10644192; doi:10.2196/49892)
Supplement: Multimedia Appendix 1 [file cardio_v7i1e49892_app1.docx]

**Multimedia Appendix 1: Question guide for three rounds of focus groups.**

Note: These are guiding questions to start the discussion. These questions are not intended to be prescriptive.

# Round 1 question guide

**Patient questions:**

1. What challenges did you face when managing your condition after hospital discharge?
2. How do you think digital technologies (e.g. Webs, apps, chatbots such as siri) can help with improving your care?
3. Have you ever used any mobile health apps, either for heart diseases, or generally?
   1. If yes, for how long? What made you continue/discontinue using mobile health apps? What could contribute to others continuing/discontinuing the use of mobile health apps?
   2. If no, why not? Why do you think people do not use mobile apps for their health, the same way that they use them for transport (such as uber), for example?
4. In your view, what are the ideal characteristics for cardiac care using technology? Would you use a technology that improves your care without the need to interact with your doctors / other health professionals?
5. What can encourage/motivate people like you to change their health behaviour in terms of physical activity, diet, and smoking?
6. What do you need the most to better follow the recommendations from your doctors/nurses?
7. How do you think that your health behaviour can be best communicated/summarised to you and then managed using information technology?

**Health professional questions:**

1. How can patients become more empowered to manage their post-mi condition after hospital discharge?
2. How can we leverage it and digital technologies to help post-mi patients change their behaviour and adopt healthier behaviours?
3. What would be the main characteristics and functions of an ideal digital solution for better management of post-mi patients?
4. Thinking of mobile apps for post-mi patients, what are the enablers and barriers for wider uptake of those apps?
5. To what extent, do you think, digital solutions should incorporate a human factor? What are the areas of patients care, if any, that can be fully automated without any involvement of healthcare providers?

**Round 2 question guide**

Note: Health professionals focus groups answered the same questions, tailored for health professionals.

# Social support

1. Would you use these features? Why/why not?
2. What would you add to these features? What would you remove?
3. How often should the online support groups run, and how long should they run for? E.g. Weekly, biweekly? Half an hour, an hour?
4. Would you be more likely to attend the online support group or in-person support group?
5. Support groups synchronous or asynchronous? I.e. Run at a certain date/time or not.
6. Should support group be text-based, audio-based, video-based?
7. Support groups moderated or not?
8. Are you likely to create a family or friend buddy? If not, would you like to have the option of an automated buddy?
9. What kind of information should the buddy access, and what can influence can they have? E.g. Notifications, nudges etc.
10. What functions can an automated buddy do? E.g. Notifications, nudges etc.

# Artificial intelligence

1. Would you use these features? Why/why not?
2. What would you add to these features? What would you remove?
3. Are you likely to try the ai chatbot? What would encourage you to use it?
4. Would you prefer if the ai chatbot used information cited from credible sources when possible?
5. What other functions can you expect from the ai chatbot? E.g. Find professionals near you, help to schedule appointments etc.
6. Which patterns of behavior would be most optimal to include in the assessment?

# Mental health

1. Would you use these features? Why/why not?
2. What would you add to these features? What would you remove?
3. How intensive do you expect the online mental health modules to be? E.g. Beginner level?
4. What key states should the online mental health address? E.g. Mood, anxiety, stress etc.
5. Would you like a timeline for when you should complete online mental health modules, or would you rather do it at your own pace?
6. The mindfulness and interoception will likely come in the form of videos or audio.

What duration would be optimal?

# Information

1. Would you use these features? Why/why not?
2. What would you add to these features? What would you remove?
3. How long should the quiz run for and what kind of questions would be most important to gauge in the quiz?
4. Do you only want information that has been tailored to you, or other more general information or information about other conditions as well (perhaps at the bottom of the page)?
5. What kind of professionals would you be interested in hearing from in the video/podcast section? E.g. Cardiologist, nutritionist etc. Duration?

# Smoking and alcohol

1. Would you use these features? Why/why not?
2. What would you add to these features? What would you remove?
3. Would you feel comfortable with the application tracking your physical location for these purposes, knowing you can turn this function on and off?
4. What kind of interventions can be delivered when the app determines you are nearing your usual smoking/drinking location?

# Round 3 question guide

In response to your suggestions about creating problem-focused behaviour change, mental health and social support tools, we have created a mental health toolkit and key information tailored for your experiences, a personal health impact page, goal setting and accomplishments medals, and a MiHeartMate buddy system with the ability to message anyone over the application.

1. In which way do you think you will use these tools to help you achieve your health goals during the mi recovery journey?

1. What are the barriers and facilitators that you anticipate may hinder / facilitate benefitting from these tools?

1. One of the main problems of digital health applications is that people tend to abandon them once the "novelty effect" is over; what other things do you think we could add / refine to stimulate you to keep using these tools for several months?

1. Are there any tools / contents that you consider irrelevant / superfluous?

Using the social outreach section as an exemplar:

(Social outreach used because it highlights several novel aspects of the app, has several new functions that weren’t discussed in the last round and has relevance to other areas of the app. E.g. MyPoints has potential to be a rewards system that other areas of the app can use, MyPeers comparison is on most pages to compare various stats. These questions also centre around making the application engaging by fostering competition and gamification which can be implemented application-wide).

1. We are looking to implement games to keep the application engaging and support your goals. One example is in social outreach, we have a MyPoints system you can play with your heartmate to earn medals, and a chat with one of our healthcare professionals – what would encourage you to use this function? How can we improve this function? Does the MyPoints system give you other ideas for how we can implement games across the application as a whole?

1. We are looking to implement a competition element (either competing with yourself, others in the app, or others in your life) to keep the application engaging and motivating. For example, in social outreach we have included a competition by comparing your statistics with other users, including the MyPeers statistics and leader board. We also give users the ability to view a leader board, follow their friends or peers on the leader board, and earn the opportunity to post a video sharing their experiences on the "sharing experiences" page. Would you use these functions and why/why not? How can we add/amend this to foster friendly and engaging competition between users? Does the MyPeers and leader board give you other ideas for how we can implement competitions across the application as a whole?

If time permits, participants can further explore the design as a group and provide further feedback.
