# Supplementary material for: Digital Health Secondary Prevention Using Co-Design Procedures: Focus Group Study With Health Care Providers and Patients With Myocardial Infarction
Source: JMIR Cardio. 2023 Oct 30;7:e49892. doi: 10.2196/49892 (PMC10644192; doi:10.2196/49892)
Supplement: Multimedia Appendix 3 [file cardio_v7i1e49892_app3.docx]

**This is a Multimedia Appendix to a full manuscript published in the J Med Internet Res. For full copyright and citation information see “Digital Health Secondary Prevention Using Co-Design Procedures: Focus Group Study With Health Care Providers and Patients With Myocardial Infarction”.**

**Appendix: Screenshots of digital health design from round 2 and 3**

Based on advice from round 1:


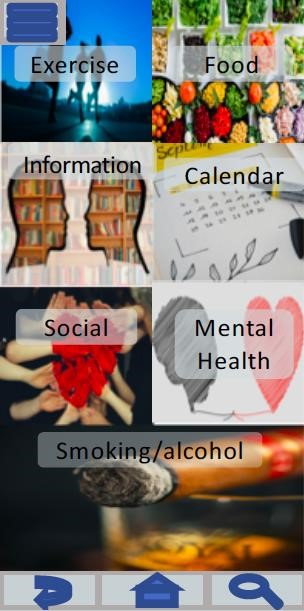

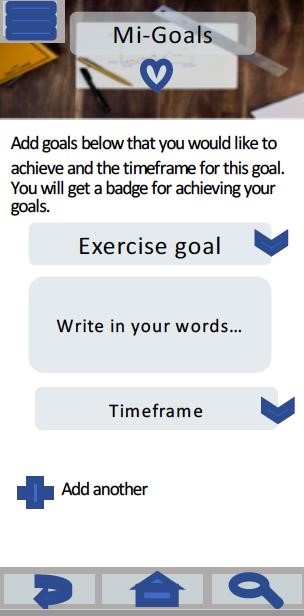

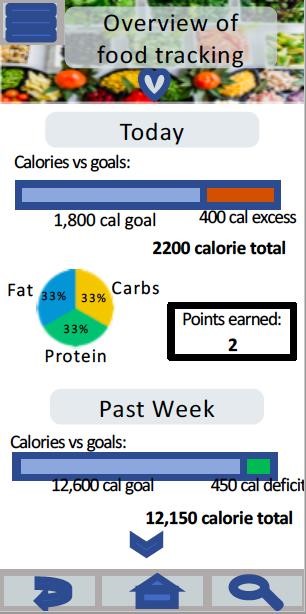

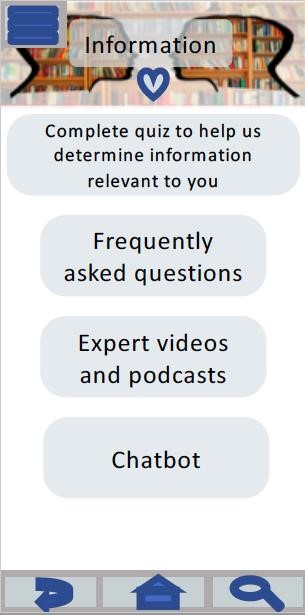

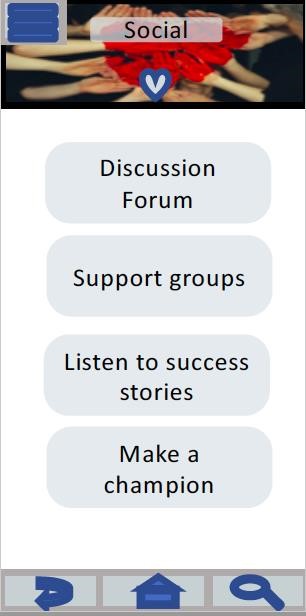

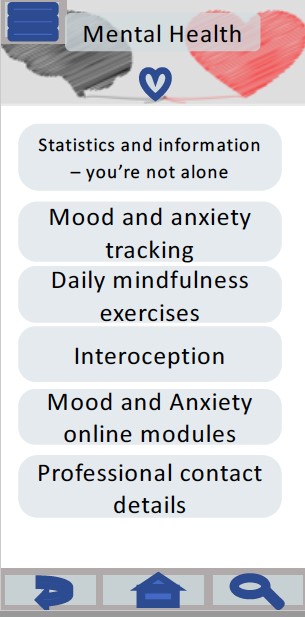


Screenshots of the MiSmartHeart conceptual design based on results from Round 1 (Consumer Needs). From left to right: home page, goal setting page, food tracking summary, information page, social support page, mental health page.

Based on advice from round 2:


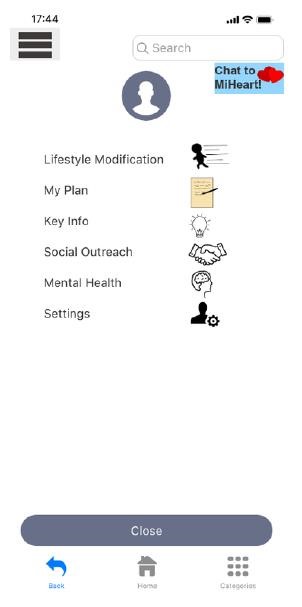

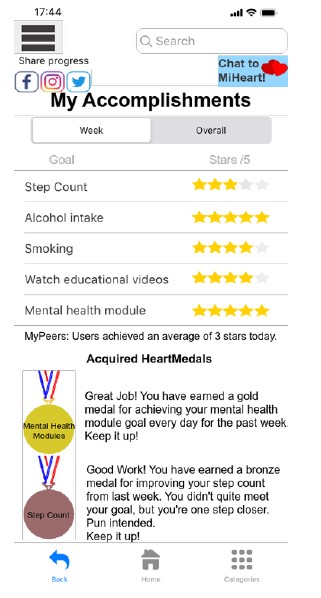

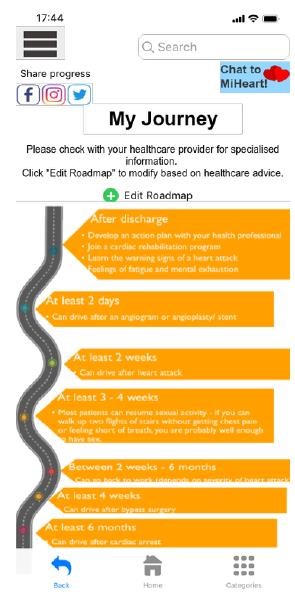

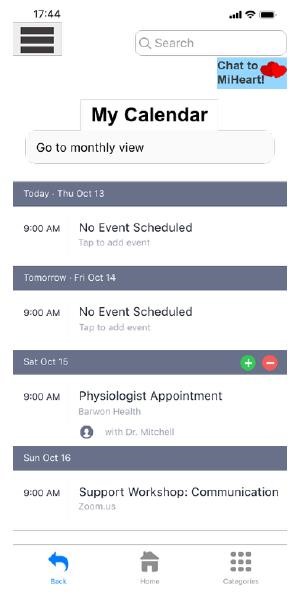

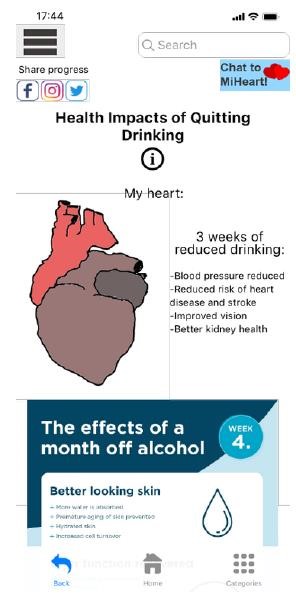

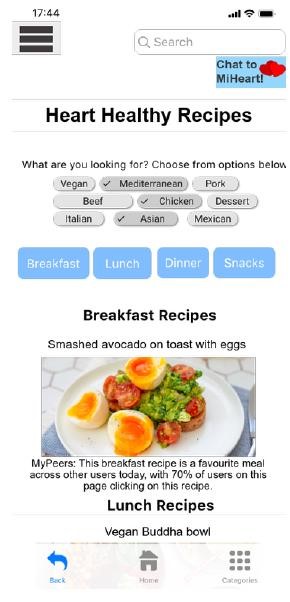


Screenshots of the MiSmartHeart simulated design based on results from round 2 (Desired Functionalities). From top left to bottom right: menu page; journey/roadmap page; calendar; accomplishments; personalized information on quitting drinking; heart healthy recipes.
